# Sign Tracking and Goal Tracking Are Characterized by Distinct Patterns of Nucleus Accumbens Activity

**DOI:** 10.1523/ENEURO.0414-18.2019

**Published:** 2019-03-15

**Authors:** Zachary S. Gillis, Sara E. Morrison

**Affiliations:** 1 Department of Neuroscience, University of Pittsburgh, Pittsburgh, PA 15260

**Keywords:** goal tracking, nucleus accumbens, Pavlovian conditioning, reinforcement learning, sign tracking

## Abstract

During Pavlovian conditioning, if a cue (e.g., lever extension) predicts reward delivery in a different location (e.g., a food magazine), some individuals will come to approach and interact with the cue, a behavior known as sign tracking (ST), and others will approach the site of reward, a behavior known as goal tracking (GT). In rats, the acquisition of ST versus GT behavior is associated with distinct profiles of dopamine release in the nucleus accumbens (NAc), but it is unknown whether it is associated with different patterns of accumbens neural activity. Therefore, we recorded from individual neurons in the NAc core during the acquisition, maintenance, and extinction of ST and GT behavior. Even though NAc dopamine is specifically important for the acquisition and expression of ST, we found that cue-evoked excitatory responses encode the vigor of both ST and GT behavior. In contrast, among sign trackers only, there was a prominent decrease in reward-related activity over the course of training, which may reflect the decreasing reward prediction error encoded by phasic dopamine. Finally, both behavior and cue-evoked activity were relatively resistant to extinction in sign trackers, as compared with goal trackers, although a subset of neurons in both groups retained their cue-evoked responses. Overall, the results point to the convergence of multiple forms of reward learning in the NAc.

## Significance Statement

An individual’s tendency to interact with a cue that predicts reward, known as sign tracking (ST), has been linked with impulsivity and addiction-related behaviors. Here, we show that, during learning, sign tracker rats, as compared with goal tracker rats, who preferentially interact with the site of reward, display different profiles of neuronal activity in the nucleus accumbens (NAc). The evolution of NAc activity is uniquely linked to the acquisition of ST, but not goal tracking (GT); however, after learning, NAc activity reflects the vigor of both behaviors. These findings imply that ST and GT result from different learning processes and engage distinct neural circuits that partially overlap in the NAc.

## Introduction

Cues that are associated with rewards, such as food or drugs, can acquire motivational value, often referred to as incentive salience (Berridge, 2004), and thereby come to exert a powerful influence over behavior. Notably, there is considerable variation among individuals in their propensity to assign incentive salience to a cue (Robinson et al., 2014). For example, in a Pavlovian conditioned approach (PCA) protocol, if a cue (e.g., extension of a lever) predicts reward in a different location (e.g., a sugar pellet delivered to a food magazine), some rats will preferentially approach and interact with the lever, a behavior known as sign tracking (ST; Hearst and Jenkins, 1974). In contrast, other rats will approach the site of reward delivery, a behavior known as goal tracking (GT; Boakes, 1977). A predisposition towards ST has been linked with measures of impulsivity (Flagel et al., 2010; Lovic et al., 2011), and susceptibility to drug-taking, addiction and relapse (Tomie et al., 2008; Saunders and Robinson, 2013).

Both ST and GT require associative learning about a cue – i.e., learning that a cue predicts reward, but only sign trackers are thought to ascribe incentive salience to the cue. Consistent with this idea, a lever cue is more effective as a conditioned reinforcer (Robinson and Flagel, 2009) and at reinstating reward-seeking behavior (Yager and Robinson, 2010) among sign trackers than among goal trackers. In fact, it has been proposed that ST and GT behaviors result from different forms of learning: one linking the cue with an explicit representation of the outcome (GT), and one linking the cue with the motivational properties of the outcome (ST; Clark et al., 2012; Huys et al., 2014; Lesaint et al., 2014). Supporting this theory, ST behavior, compared with GT, is resistant to changes in the cue-outcome relationship, including reward devaluation (Cleland and Davey, 1982; Morrison et al., 2015; although see Derman et al., 2018) and extinction (Ahrens et al., 2016).

Many studies have shown that mesolimbic structures such as the nucleus accumbens (NAc), and especially dopamine therein, play an essential role in conditioned approach, including ST. Lesions of the NAc core impair PCA and produce deficits in the acquisition and expression of ST (Cardinal et al., 2002; Chang et al., 2012; although see Chang and Holland, 2013); moreover, NAc dopamine depletion (Parkinson et al., 2002) or receptor blockade (Flagel et al., 2011; Saunders and Robinson, 2012) reduce ST while affecting GT minimally or not at all. Similarly, injection of amphetamine into the NAc increases ST but not GT (Singer et al., 2016). Furthermore, both sign tracker and goal tracker individuals exhibit phasic dopamine release in the NAc in response to reward-predictive cues; however, only sign trackers show increasing dopamine release in response to the cue and decreasing dopamine release in response to the reward over the course of training (Flagel et al., 2011). This finding implies that acquisition of ST, but not GT, requires a form of learning that depends on the reward prediction error encoded by mesolimbic dopamine.

Although sign trackers and goal trackers exhibit different characteristic profiles of NAc dopamine release (Flagel et al., 2011), it is unclear whether and how these differences impact NAc neuronal activity supporting different forms of learning. In order to address this question, we recorded the activity of individual neurons in the NAc core during the acquisition, maintenance, and extinction of ST and GT behaviors. Studies using instrumental tasks have shown that cue-evoked firing in the NAc encodes both the reward associations of the cue and the vigor of the subsequent locomotor response (McGinty et al., 2013; Morrison et al., 2017). Therefore, we hypothesized that NAc activity would reflect the vigor of both ST and GT behaviors. Alternatively, robust differences in the representation of the locomotor properties of ST versus GT might indicate a preferential role for NAc activity in promoting one of these behaviors.

At the same time, we anticipated that different patterns of task-related activity would emerge in sign tracker versus goal tracker individuals, reflecting the different learning processes – a dopamine-dependent form of learning resulting in ST, and a dopamine-independent form of learning resulting in GT – that have been predicted to converge in the NAc (Clark et al., 2012; Lesaint et al., 2014). On the other hand, if we did not find such a dissociation, it would raise new questions regarding the functional relevance of differences in NAc dopamine release during the acquisition of ST and GT.

## Materials and Methods

All animal procedures were performed in accordance with the University of Pittsburgh animal care committee’s regulations.

### Subjects

Subjects were eight male Long–Evans rats obtained from Charles River Laboratory weighing 275–300 g upon arrival. Rats were pair-housed until surgery (see below) on a 12/12 h light/dark cycle (lights on at 7 P.M.). All experiments were performed during the dark phase. After arrival, rats were allowed to acclimate to the housing colony for 7 d. They were then habituated to human contact and handling over at least two sessions prior to surgery and the start of behavioral training. Subjects were provided with water *ad libitum* throughout and food *ad libitum* until 2 d before the start of training, when they were placed on a restricted diet of 15 g of chow per day. Rats were weighed regularly, and, if necessary, provided with extra food to maintain a minimum of 90% of pre-restriction body weight.

### Implantation of electrode arrays

Using standard aseptic procedures, we implanted custom-constructed fixed electrode arrays bilaterally targeted at the NAc core (coordinates in mm from bregma: AP = +1.4, ML = ±1.5, DV = –7.0 from dura). Recording arrays comprised eight Teflon-insulated tungsten wires (A-M Systems) hand-cut to achieve an impedance of 90–110 MΩ and mounted in a circular pattern (diameter ∼1 mm). Animals were anesthetized with isoflurane (4% for induction, 1–2% for maintenance) and treated with ketoprofen (5 mg/kg) for pain relief, as well as acetaminophen in their drinking water for 3 d following surgery. Animals were allowed to recover for at least 7 d prior to food restriction and the start of behavioral training.

### Histology

After completion of data collection, animals were deeply anesthetized using chloral hydrate (400 mg/kg) and direct current (75 µA) passed through each of the electrodes in the array for 10 s. Animals were then transcardially perfused with saline followed by 10% buffered formalin; brains were removed and placed in formalin. Brains were sunk in 30% sucrose for at least 3 d before sectioning on a cryostat (60-µm slices), followed by staining with cresyl violet. Placement of electrode arrays was confirmed via light microscope.

### Apparatus and behavior

All training and experiments took place in a standard operant chamber (Coulbourn Instruments) equipped with a house light, a speaker for auditory cues, and a pellet dispenser connected to a food magazine recessed into the side wall. The magazine was equipped with an infrared photo-detector unit to detect entries and exits. Two retractable levers were installed on either side of the magazine, although only one lever (counterbalanced among subjects) was used for each subject. White cue lights were present above each lever. The behavioral task was controlled by Coulbourn software (GraphicState 3.0).

Rats were trained using a PCA procedure similar to those used previously (Morrison et al., 2015; Tunstall and Kearns, 2015). Each training session began with illumination of the house light. Rats were initially trained over two sessions to retrieve sugar pellets (45 mg, Bio-Serv) from the magazine, with each session consisting of 50 rewards delivered on a variable interval schedule averaging 60 s. During the second magazine training session, rats were habituated to the recording apparatus (see below).

Following magazine training, subjects completed seven consecutive daily acquisition sessions on the PCA task. Neuronal recording took place on all 7 d. The PCA task consisted of 25 trials separated by an intertrial interval selected from a truncated exponential distribution averaging 60 s. Each trial was initiated by the presentation of the cue: lever extension accompanied by a brief auditory stimulus (1 s, 500-Hz intermittent tone) and flashing of the corresponding cue light (5 Hz). After 8 s, the lever retracted, the cue light extinguished, and the reward was delivered into the magazine. No action was required for reward to be delivered.

In a subset of subjects (*n* = 7), rats were subsequently retrained for 1 d, followed by an extinction procedure, which was identical to the PCA task except that no reward was delivered. The lag between the last acquisition session and retraining/extinction ranged from 5 to 14 d. No substantive differences in behavior or neural responses were seen in the groups that underwent extinction earlier versus later, so data were combined for subsequent analysis.

### Electrophysiology

We recorded neuronal activity throughout task acquisition, maintenance, and extinction using Plexon hardware and software. Rats were connected to a light-weight headstage and a motorized commutator that allowed free movement. Voltages were bandpass filtered between 220 Hz and 6 kHz, amplified 500×, and digitized at 40 kHz. Putative spikes were time-stamped and stored in segments of 1.4 ms, followed by sorting (Offline Sorter, Plexon) using principal component analysis and visual inspection of waveform clusters in 3D feature-based space. Only units with a peak amplitude >75 µV, as signal-to-noise ratio exceeding 2:1, and fewer than 0.1% of interspike intervals <2 ms were analyzed. We verified isolation of single units by inspecting autocorrelograms, as well as cross-correlograms for those units recorded on the same electrode.

### Analysis of behavior

All analyses were carried out using custom-written programs in MATLAB. We quantified the degree to which rats engaged in ST and GT by calculating a PCA index (Meyer et al., 2012; Morrison et al., 2015), which comprises the average of three ratios: (1) a probability index, which compares the probability of lever deflection versus magazine entry during the 8-s cue, calculated as (P_lever_ – P_magazine_), (2) a bias index, which compares the average number of lever deflections and magazine entries per cue, calculated as (#lever – #magazine)/(#lever + #magazine), and (3) a latency index, which compares the average latency from cue onset to lever deflection versus latency from cue onset to magazine entry, calculated as (magazine latency – lever latency)/(cue length). For trials in which a behavior was not performed, the latency for that behavior was defined as the cue length (8 s). All of these indices, including the PCA index, range from –1.0 to +1.0, with more positive numbers for animals that preferentially sign track (interact with the lever) and more negative numbers for animals that preferentially goal track (interact with the magazine). Sign trackers were operationally defined as those subjects with PCA index greater than the mean PCA index on the final day of training; all other subjects were categorized as goal trackers.

Two subjects (both goal trackers) were not included in the dataset for the first day of training because a software error rendered the recording inaccessible. One subject was not included in the dataset for the last day (day 7) of training because no neurons could be isolated during that session; for the same reason, this subject did not undergo extinction and was therefore not included in the extinction dataset.

### Analysis of neural activity

To identify neurons with excitatory responses to the cue, we used a Poisson distribution to approximate the baseline firing rate of each recorded cell during the 1 s prior to cue onset. Cue-excited neurons were identified as such by the presence of three consecutive 10-ms bins within the 500 ms after cue onset in which firing rate exceeded the 99.9% confidence interval of the baseline distribution. We also examined whether the cue response was primarily excitatory or inhibitory by calculating the mean Z-score relative to baseline in 10-ms bins over the 200 or 400 ms following cue onset. If this value was negative for both bins, the neuron was excluded from analysis. Finally, we excluded from analysis a handful of neurons with baseline firing rates too low (<0.5 Hz) to ensure isolation throughout the session.

Responses to reward delivery were identified in a similar manner to cue responses, except that the Poisson distribution was fit to firing rate during the 1 s prior to reward delivery. Excitatory and inhibitory responses were identified by the presence of three consecutive 10-ms bins within the 500 ms after reward delivery in which firing rate exceeded the upper 99.9% confidence interval or was less than the lower 99.9% confidence interval, respectively.

To evaluate whether individual neurons remained stable across sessions, we first identified a subset of candidate units that were present on all seven training days, and then applied a simple waveform similarity analysis (Kennedy and Shapiro, 2009). Briefly, for each neuron’s waveform, we calculated the daily average voltage deflection at peak and trough, and computed the Pearson’s correlation coefficient (*r*) for peak and trough across days. Units with |r| > 0.9 and *p* < 0.05 were considered stable. Because many recorded neurons did not meet these criteria, and many more were not present for all 7 d of recording, we did not perform analyses that would rely on neuronal stability (other than the examples shown in Extended Data Fig. 5-1).

Peristimulus time histograms (PSTHs) for individual neurons were calculated in 10-ms bins and are shown smoothed with a 5-bin moving average. Population PSTHs were also calculated in 10-ms bins and normalized relative to a 1-s pre-cue baseline before averaging across neurons. The average activity was smoothed for display using a 5-bin moving average.

Analyses were performed on firing rates from a 500-ms window following cue onset or reward delivery unless otherwise specified. In cases where an alternate window of 1 s was used, results did not qualitatively differ when data were reanalyzed using a 500-ms window. In some cases, we used ROC analysis to generate an “index” to compare two distributions of firing rates. For these indexes, which are derived from the area under the ROC curve, a value of 0.5 indicates that the two distributions are indistinguishable. To generate *p* values for individual indexes, we performed permutation tests by randomly reshuffling the data 1000 times.

Within extinction sessions, we identified cue-excited neurons that decreased their cue-related activity over the course of the session using a one-way ANOVA with trial number as a continuous variable. If the *p* value was <0.01 for firing rate in either a 200- or 500-ms window after cue onset and activity decreased over the course of the session, the neuron was categorized as an “extinguishing” cell. Only one cell significantly increased its activity over the course of the session and was excluded from further analysis. The remaining neurons were categorized as “non-extinguishing” cells.

## Results

**Figure 1.**
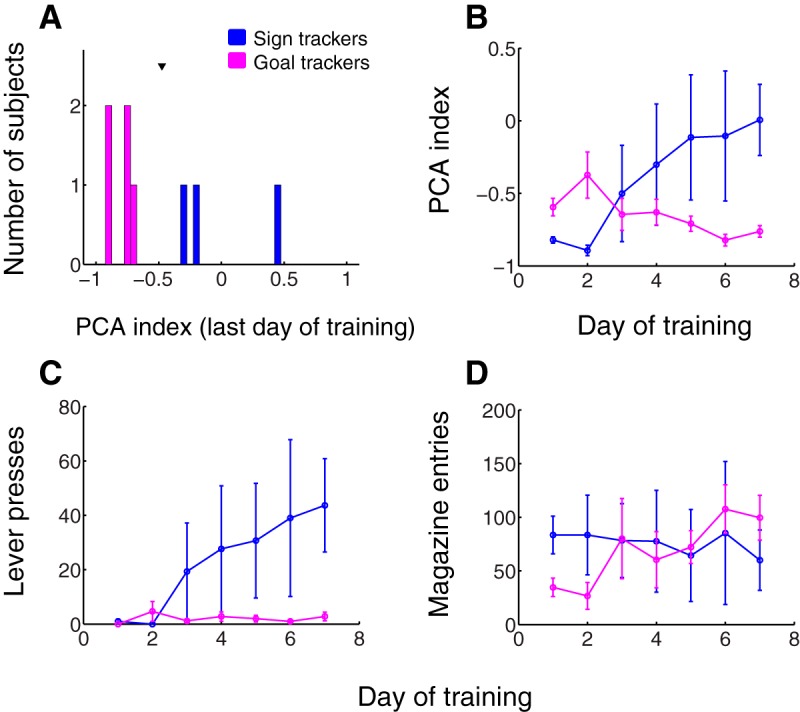
Sign tracker and goal tracker individuals differed mainly in their level of interaction with the lever cue. ***A***, PCA index (see Materials and Methods) for all subjects measured during the last training session (day 7). Arrowhead, mean PCA index. Blue, subjects categorized as sign trackers; magenta, goal trackers. ***B–D***, PCA index (***B***), total lever presses (***C***), and total magazine entries during the cue (***D***) over all 7 d of training for sign trackers (blue) and goal trackers (magenta). Error bars, SEM.

We used fixed electrode arrays to record from individual neurons in the NAc core while rats (*n* = 8) acquired and performed a PCA task similar to others that have been used to study ST and GT behavior (Meyer et al., 2012; Morrison et al., 2015; Tunstall and Kearns, 2015). In this task, ST is represented by lever presses and GT is represented by entries into a food magazine. We quantified individual rats’ propensity towards ST and GT behavior by calculating a PCA index (Meyer et al., 2012) that ranges from –1.0 (all GT, no ST) to +1.0 (all ST, no GT). On the last day of training (day 7), subjects exhibited a wide range of ST and GT behavior; however, all rats performed some degree of GT, resulting in a PCA index distribution that was negatively skewed (Fig. 1*A*). Therefore, we divided subjects into “sign trackers” (STs) and “goal trackers” (GTs) based on whether each individual’s PCA index on the last day of training was above or below the mean. This definition categorized as STs only those subjects with an appreciable degree of interaction with the lever. Indeed, we observed that operationally defined STs behaved in a qualitatively different manner from GTs, with marked orienting towards the lever and sniffing, biting, and gnawing behaviors directed towards the lever.

In agreement with previous studies (Morrison et al., 2015), sign trackers’ PCA index steadily increased over the course of training while that of goal trackers stayed the same or decreased slightly (Fig. 1*B*). This was largely driven by a robust increase in the number of lever presses by sign trackers (Fig. 1*C*) while all subjects’ magazine entries during the cue remained relatively stable (Fig. 1*D*), with only a small increase in entries for goal trackers and decrease in entries for sign trackers over the seven sessions.

### NAc cue-evoked activity encodes the vigor of subsequent ST and GT behavior

**Figure 2.**
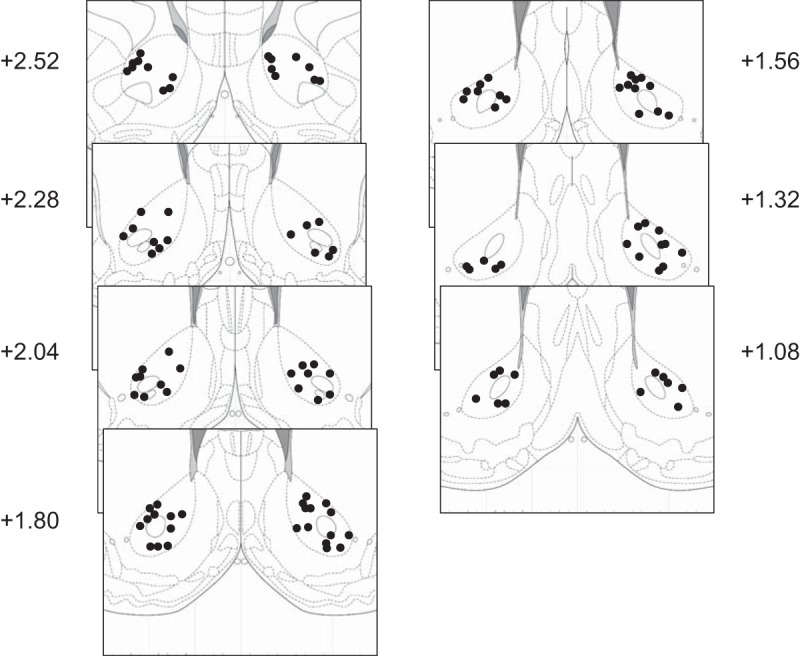
Histological reconstruction of recording locations in NAc core. Panels are coronal atlas sections (Paxinos and Watson, 2007) showing the location of electrode tips derived from electrolytic lesions and/or electrode tracks. Numbers are distance in millimeters from bregma.

We recorded from 122 individual neurons on the final day of training; recording locations based on histological reconstruction are shown in Figure 2. Of these neurons, approximately half (58/122; 47.5%) exhibited excitatory responses evoked by cue onset, consistent with prior reports from studies using instrumental tasks (McGinty et al., 2013; Morrison and Nicola, 2014; Morrison et al., 2017). Of these, 15 cells were recorded from sign tracker individuals (*n* = 3) and 43 from goal tracker individuals (*n* = 4). One subject did not contribute to data from the final day of training because no cells could be isolated during that session. There were no obvious differences in firing characteristics in cells recorded from sign trackers versus goal trackers; their baseline firing rates were statistically identical (*p* = 0.7, Wilcoxon rank-sum test).

**Figure 3.**
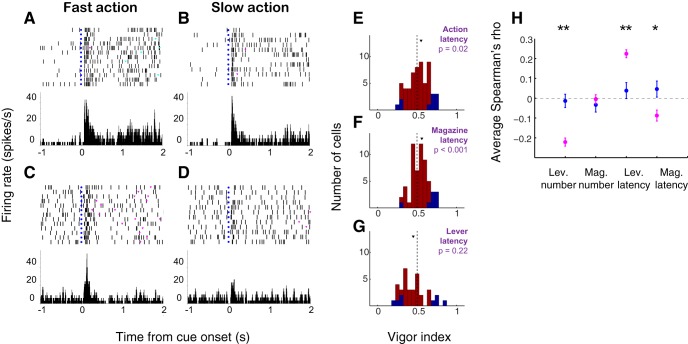
The vigor of both ST and GT behavior may be represented in NAc firing. ***A***, ***B***, Example of a neuron with stronger cue-evoked excitation when the cue is followed by a lever press with shorter (***A***) rather than longer (***B***) latency. ***C***, ***D***, Example of a neuron with stronger cue-evoked excitation when the cue is followed by a magazine entry with shorter (***C***) rather than longer (***D***) latency. Left panels, action latency <50th percentile; right panels, action latency ≥50th percentile. Trials are shown in chronological order with earliest on top. Blue dots, cue onset; magenta triangles, magazine entries; cyan triangles, lever presses. ***E–G***, On the population level, representation of latency to magazine entry (i.e., GT) predominates. ***E***, Vigor index for latency to first action after cue onset. Median index is greater than 0.5 (*p* = 0.02, Wilcoxon). ***F***, Vigor index for latency to magazine entry. Median index is greater than 0.5 (*p* < 0.001, Wilcoxon). ***G***, Vigor index for latency to lever press. Distribution not different from 0.5 (*p* = 0.22, Wilcoxon signed-rank test). All panels, blue indicates significant vigor index (*p* < 0.05, permutation test); arrowhead indicates median. ***H***, Average Spearman’s rank correlation coefficient (rho) between cue-evoked neural activity in the 500 ms following cue onset and the indicated behavioral measure for sign trackers (blue) and goal trackers (magenta). From left to right: number of lever presses, number of magazine entries, latency to first lever press, latency to first magazine entry. Single asterisk, *p* < 0.05, Wilcoxon rank-sum test; double asterisk, *p* < 0.001. Distribution of individual correlation coefficients may be found in Extended Data Fig. 3-1.

It has previously been observed that cue-evoked excitations in the NAc encode the vigor, including latency and speed, of subsequent approach to a target during instrumental tasks, as well as information about whether the target is associated with a reward (McGinty et al., 2013; Morrison et al., 2017). Because the NAc is also essential for PCA (Day and Carelli, 2007), and for ST behavior in particular (Cardinal et al., 2002; Chang et al., 2012), we examined whether NAc cue-evoked activity similarly encodes the vigor of approach in a Pavlovian context, and whether this encoding differs for ST versus GT behavior. Indeed, we noted that many individual neurons responded more strongly to the cue when the subsequent behavior was faster or more vigorous. For example, Figure 3*A*,*B* shows a neuron recorded in a sign tracker subject that had stronger cue-evoked firing when the cue was followed by a lever press with short latency; Figure 3*C*,*D* shows a different neuron, from a goal tracker subject, that had stronger cue-evoked firing when the cue was quickly followed by a magazine entry.

In order to quantify this effect throughout the population, we calculated a “vigor index” using ROC analysis (see Materials and Methods) that compared the magnitude of cue-evoked excitations on trials with relatively short latency versus long latency to action. A vigor index greater than 0.5 indicates higher firing when the subsequent action occurred with shorter latency; an index less than 0.5 indicates higher firing when the subsequent action occurred with longer latency. When evaluated on a cell-by-cell basis, the distribution of the vigor index for latency to first action (either lever press or magazine entry) was significantly shifted to the right of 0.5 (Wilcoxon signed-rank test, *p* = 0.02; Fig. 3*E*), indicating stronger neural responses prior to short-latency actions. Notably, the vigor of GT was encoded more robustly than that of ST: when the vigor index was calculated for latency to magazine entry, the resulting distribution was significantly shifted from 0.5 (*p* < 0.001; Fig. 3*F*), whereas the vigor index for latency to lever press was not different from 0.5 when evaluated across the whole population of neurons (*p* = 0.22; Fig. 3*G*). This was the case for sign tracker and goal tracker subjects considered separately as well as together.

We next examined whether NAc neural activity is related to the expression of ST and/or GT behavior on a trial-by-trial basis. To do so, we calculated the Spearman’s rank correlation coefficient (rho) for each cell between firing rate (500-ms window after cue onset) and the magnitude or latency of behavior over the last 2 d of training (50 trials). Many individual correlations were significant (Extended Data Fig. 3-1), especially among goal trackers, who exhibited neural activity that was positively correlated with the vigor of magazine entry and negatively with the vigor of lever pressing. The average Spearman’s rho for each behavioral measure is shown in Figure 3*H*. Overall, neurons recorded in goal trackers had significantly larger correlation coefficients for most behaviors, including latency to first magazine entry (*p* = 0.007, Wilcoxon rank-sum test), as well as lever press number and latency (*p* < 0.001 for each), but, interestingly, not number of magazine entries (*p* = 0.75). Meanwhile, the activity of neurons recorded in sign trackers, although they sometimes varied with behavior on an individual basis (Extended Data Fig. 3-1), did not show correlations that were significantly different from zero, on average (*p* > 0.2 for all measures, Wilcoxon signed-rank test).

10.1523/ENEURO.0414-18.2019.f3-1 Extended Data Figure 3-1 Correlation of the activity of individual neurons with trial-by-trial ST and GT behavior. Distribution of Spearman’s rank correlation coefficient (rho) relating cue-evoked neural activity (500-ms window) to number of lever presses (***A***, ***B***), number of magazine entries (***C***, ***D***), latency to first lever press (***E***, ***F***), or latency to first magazine entry (***G***, ***H***) for individual neurons recorded in sign trackers (***A***, ***C***, ***E***, ***G***) or goal trackers (***B***, ***D***, ***F***, ***H***) over the last 2 d of training. All panels, blue indicates significant correlation (α = 0.1), and *p* values indicate results of Wilcoxon signed-rank test for median different from zero. Download Figure 3-1, EPS file.

Overall, even though ST and GT are thought to represent the output of separate learning processes that engage different neural circuits (Lesaint et al., 2014), the vigor of each behavior – and, surprisingly, GT even more than ST – is represented by a subset of cue-excited neurons in the NAc. This is consistent with the proposed role of the NAc as a node of interaction for multiple brain systems that promote approach towards a reward-associated target (Nicola, 2010; Clark et al., 2012).

### NAc activity evolves differently in sign tracker and goal tracker individuals over the course of behavior acquisition

Although it has been established that ST and GT individuals develop distinct patterns of NAc dopamine release over the course of learning (Flagel et al., 2011), it remains unclear whether and how this corresponds with differences in the activity of single neurons. Therefore, we next asked how NAc activity changes with respect to task events during early and late stages of acquisition of ST and GT behavior.

**Figure 4.**
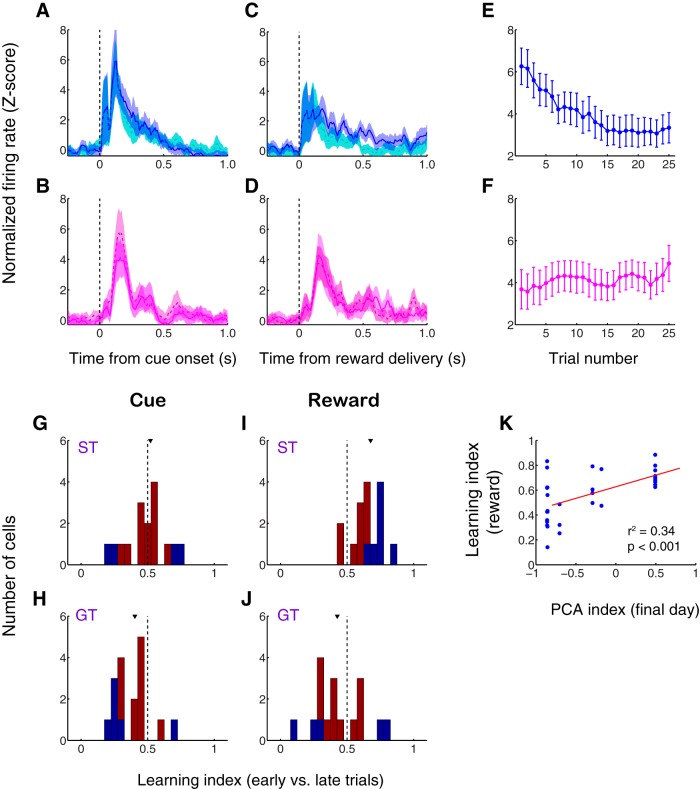
Sign trackers and goal trackers exhibit differences in NAc activity on the first day of training. ***A–D***, Population average normalized activity aligned on cue onset (***A***, ***B***) or reward delivery (***C***, ***D***) for sign tracker (***A***, ***C***) and goal tracker (***B***, ***D***) subjects. Blue and magenta solid lines, first half of trials (trials 1–12); cyan and pink dashed lines, second half of trials (trials 13–25). Shading, SEM. ***E***, ***F***, Trial-by-trial normalized activity in response to reward delivery (1-s window) for sign trackers (***E***) and goal trackers (***F***). Error bars, SEM. ***G–J***, Distribution of learning index for sign trackers (***G***, ***I***) and goal trackers (***H***, ***J***) derived from ROC analysis comparing the first half and second half of trials. Index >0.5 indicates higher cue-evoked (***G***, ***H***) or reward-evoked (***I***, ***J***) activity during early trials. Blue represents index significantly different from 0.5 (*p* < 0.05, permutation test). Arrowheads indicate median. The median is significantly greater than 0.5 for reward-evoked activity in sign trackers only (*p* < 0.001, Wilcoxon signed-rank test). ***K***, PCA index for behavior from the final day of training plotted against the learning index for reward-related neural activity (1-s window). Regression line in red.

Starting with the first day of training on the PCA task, we found clear differences between sign trackers and goal trackers in the evolution of NAc activity. We recorded from 64 individual neurons in six subjects during day 1 of training; of these, 33 cells (51.6%) exhibited cue-evoked excitatory responses, 16 of which were recorded from sign tracker subjects and 17 from goal trackers. In most cases, cue-evoked excitations were present on the very first training trial. In order to examine how neural responses changed over the course of the session, we divided the session into “early trials” (trials 1–12) and “late trials” (trials 13–25). On a population level, there was no significant difference in firing in the 500 ms after cue onset during early versus late trials in either sign trackers (*p* = 0.08, Wilcoxon rank-sum test) or goal trackers (*p* = 0.37; Fig. 4*A*,*B*). Moreover, cue-evoked activity was slightly higher in sign trackers than in goal trackers during early trials (*p* = 0.01, Wilcoxon rank-sum test), and indistinguishable between the two groups during later trials (*p* = 0.5).

In contrast, in sign trackers only, there was a significant decrease in firing in the 500 ms following reward delivery during the first half versus the second half of trials (*p* < 0.001, Wilcoxon rank-sum test; Fig. 4*C*). In goal trackers, on the other hand, population-level reward-related activity remained stable over the course of the training session (*p* = 0.18; Fig. 4*D*). Similarly, during the first half of trials, reward-related activity was slightly higher in sign trackers than in goal trackers (*p* = 0.02, Wilcoxon rank-sum test); however, during the second half of trials, reward-related activity in sign trackers decreased to a level significantly below that of goal trackers (*p* = 0.006). This pattern was also apparent when we examined reward-related responses on a trial-by-trial basis: median reward-evoked firing during the first five trials of the session was significantly greater than firing during the last five trials in sign trackers (*p* < 0.001, Wilcoxon rank-sum test; Fig. 4*E*) but not in goal trackers (*p* = 0.07; Fig. 4*F*).

In order to quantify this effect on a cell-by-cell basis, we calculated a “learning index” based on ROC analysis (see Materials and Methods) that compared the magnitude of cue-evoked responses (Fig. 4*G*,*H*) or reward-evoked responses (Fig. 4*I*,*J*) during the first half and second half of trials. A learning index value greater than 0.5 indicates higher firing during early trials – i.e., decreasing activity over the course of the session – while an index less than 0.5 indicates higher firing during late trials: i.e., increasing activity over the course of the session. Among sign trackers, the median learning index for cue-evoked activity was not different from 0.5 (*p* = 0.82, Wilcoxon signed-rank test), whereas the median for reward-evoked activity was significantly greater than 0.5 (*p* < 0.001), indicating that a substantial proportion of individual neurons showed decreasing reward-related responses over the course of the session. Among goal trackers, on the other hand, the median learning index for cue-evoked activity (Fig. 4*H*) was slightly less than 0.5 (*p* = 0.01), reflecting a small increase in firing in the 1 s following the cue, but the median learning index for reward-related activity was not different from 0.5 (*p* = 0.29).

Consistent with the above results, we found that the learning index for reward-related activity was markedly higher in sign trackers than in goal trackers (*p* < 0.001, Wilcoxon rank-sum test); in contrast, the learning index for cue-related activity was slightly higher in sign trackers (*p* = 0.05), which can be entirely attributed to the small increase in cue-evoked activity among goal trackers over the first day of training. Finally, an individual subject’s relative degree of ST versus GT behavior on the last day of training, represented by the PCA index, was significantly correlated with the learning index for reward-related activity observed in cells recorded from that subject (*r* ^2^ = 0.34, *p* < 0.001; Fig. 4*K*). Thus, during the first training session, cue-evoked activity showed only minor changes or no changes in both sign trackers and goal trackers, whereas reward-related activity exhibited a significant decrease over the course of the session in sign trackers only – a decrease that was markedly more robust in those individuals with the greatest tendency to sign track later on.

It has been shown that, among outbred rats with a propensity for ST, cue-evoked NAc dopamine release increases, and reward-evoked dopamine release decreases, over the course of 6 d of training (Flagel et al., 2011). The same was not true of outbred rats that were categorized as goal trackers. In light of this finding, we wished to examine whether NAc cue-evoked and/or reward-evoked neuronal activity differs between sign trackers and goal trackers on the last day of training, in parallel with dopamine release, and whether these groups show differences in the evolution of their task-related neuronal firing over the full course of training.

**Figure 5.**
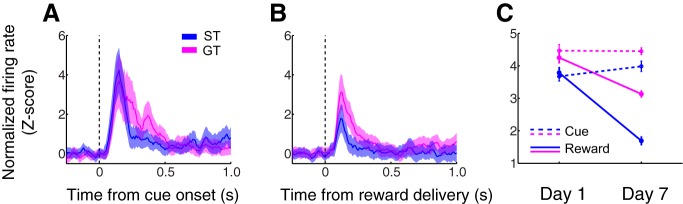
Sign trackers exhibit an attenuated reward response on the last day of training. ***A***, ***B***, Population average normalized activity aligned on cue onset (***A***) or reward delivery (***B***). Shading, SEM. Individual cell examples may be found in Extended Data Fig. 5-1. ***C***, Population-wide average neural activity in the 1 s following cue onset (dashed lines) or reward delivery (solid lines) on the first day of training (left) and the last day of training (right). Error bars, SEM. All panels: sign trackers in blue, goal trackers in magenta.

We found that, after behavior was fully established, sign trackers and goal trackers showed only minor differences in cue-evoked firing, but diverged markedly in their response to reward. Indeed, on the final day of training, there was no significant difference on a population level between sign trackers and goal trackers in firing in the 1-s window after cue onset (*p* = 0.52, Wilcoxon rank-sum test; Fig. 5*A*). In contrast, activity in the 1 s following reward delivery was significantly diminished in sign trackers relative to goal trackers (*p* < 0.001; Fig. 5*B*). The majority of cue-excited cells were also excited by reward (38 out of 58); of the remaining cue-excited cells, eight were reward-inhibited, and 12 had no significant response to reward delivery. Some of these 20 cells may have decreased their reward response over the course of training; consistent with such decreases being more prevalent in sign trackers, a disproportionate number of these were found in sign trackers, although the disparity was just short of reaching significance (*p* = 0.07, χ^2^ test).

10.1523/ENEURO.0414-18.2019.f5-1 Extended Data Figure 5-1 Evolution of cue-related and reward-related activity over the course of training in two example neurons. Two representative neurons with highly stable waveforms over the course of training (seven sessions) were selected for analysis, one from a sign tracker (***A***, ***B***) and one from a goal tracker (***C***, ***D***). ***A***, ***C***, Heat plots show activity related to the cue (left) or reward (right) during each of the seven training sessions as average firing rates calculated in 10-ms bins with no smoothing. ***B***, ***D***, Average firing rate over the 500 ms following cue onset (dashed lines) or reward delivery (solid lines) for the cells shown in ***A***, ***C***, respectively. Error bars, SEM. Download Figure 5-1, EPS file.

We next assessed how task-related activity, on a population level, evolved over the full course of training. Examining activity in a 1-s window following either cue onset or reward delivery (Fig. 5*C*), we found that subjects’ cue-evoked excitatory responses remained stable, on average, between day 1 and day 7 of training (sign trackers, *p* = 0.31; goal trackers, *p* = 0.22, Wilcoxon rank-sum test). In contrast, reward-related firing decreased significantly among both sign trackers and goal trackers (both, *p* < 0.001) from day 1 to day 7, with a more dramatic decrement in activity averaging –55% in sign trackers (compared to –26% in goal trackers). Although we had no definitive way to assess whether the same cells were recorded from day to day, we used a simple waveform similarity analysis (see Materials and Methods), to identify a small number of individual neurons that appeared to be stable across all 7 d. Two representative examples, one each from a sign tracker and a goal tracker, are shown in Extended Data Figure 5-1. The activity of these two neurons reflects the same trends as the overall population average. Overall, these data support the observation that reward-related activity, but not cue-related activity, in the NAc core decreases in prominence over the course of training, a decrease that is more robust in sign trackers than in goal trackers and that is apparent whether activity is sampled at an early or late stage of training.

### Distinct patterns of NAc cue-evoked activity and behavior during extinction among sign tracker and goal tracker individuals

It has previously been shown that ST behavior, compared to GT behavior, is relatively impervious to changes in the cue-outcome relationship, including both reward devaluation (Morrison et al., 2015) and extinction (Ahrens et al., 2016). Because it is thought that NAc activity plays an important role in promoting Pavlovian approach (Cardinal et al., 2002; Day and Carelli, 2007; Morrison and Nicola, 2014), including ST, we next asked whether NAc cue-evoked excitations “extinguish” in concert with behavior in the current task. We therefore exposed a subset of subjects (*n* = 7; three sign trackers and four goal trackers) to a single extinction session following the completion of training on the PCA task; the extinction procedure was identical to the PCA task except that no rewards were delivered. We chose to carry out the extinction session on a separate day from training in order to ensure that the subject’s behavioral state was comparable to previous sessions (i.e., by removing the possible confounds of satiety, boredom, or fatigue.) During extinction, we recorded from 78 individual neurons, of which 53 (68.0%) exhibited cue-evoked excitatory responses, 17 from subjects categorized as sign trackers and 36 from goal trackers.

**Figure 6.**
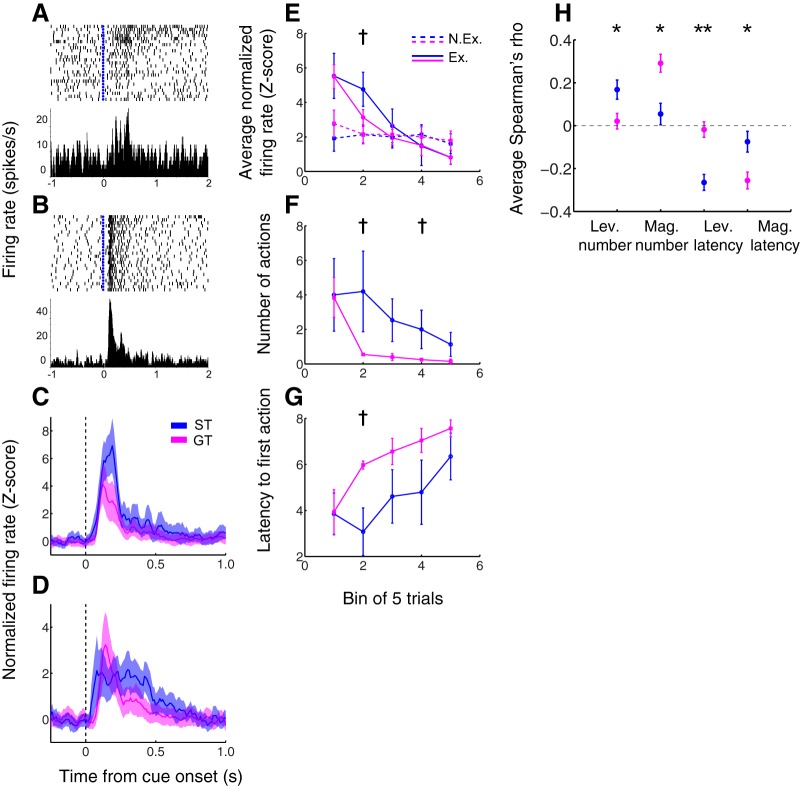
In sign trackers, as compared to goal trackers, behavior and cue-evoked firing are resistant to extinction. ***A***, ***B***, Two example neurons recorded during the same extinction session. Within the same subject, some NAc neurons extinguish their cue-evoked firing (as in ***A***), and some do not (as in ***B***). Trials are shown chronologically with the earliest trial on top. Blue dots, cue onset. ***C***, ***D***, Population average activity during extinction sessions for extinguishing cells (***C***) and non-extinguishing cells (***D***). Shading, SEM. ***E***, Cue-evoked neural responses in the 500 ms after cue onset for extinguishing cells (Ex.; solid lines) and non-extinguishing cells (N.Ex.; dashed lines). Activity is averaged in five-trial bins. ***F***, ***G***, Average behavior during extinction sessions for sign trackers (blue; lever presses only) and goal trackers (magenta; magazine entries only). The number (***E***) and latency (***F***) of actions are averaged in five-trial bins. Blue, sign trackers; magenta, goal trackers. All panels, dagger indicates *p* < 0.1, Wilcoxon rank-sum test. ***H***, Average Spearman’s rank correlation coefficient (rho) between cue-evoked neural activity in the 500 ms following cue onset and the indicated behavioral measure for sign trackers (blue) and goal trackers (magenta). All comparisons between sign trackers and goal trackers are significant. From left to right: number of lever presses, number of magazine entries, latency to first lever press, latency to first magazine entry. Single asterisk, *p* < 0.05, Wilcoxon rank-sum test; double asterisk, *p* < 0.001. Distribution of individual correlation coefficients may be found in Extended Data Fig. 6-1.

We found that many individual neurons in the NAc indeed exhibit reductions in cue-evoked firing over the course of an extinction session; in some cases, the cue-evoked excitation is entirely absent by the end of the session. Intriguingly, however, other individual neurons, often within the same subject, exhibit no apparent decrease in cue-evoked firing over the course of extinction. To quantify this phenomenon, we used a one-way ANOVA with trial number as a continuous factor (see Materials and Methods) to categorize neurons as extinguishing or non-extinguishing. Figure 6*A*,*B* shows a representative example of two neurons, one extinguishing cell (Fig. 6*A*) and one non-extinguishing cell (Fig. 6*B*), recorded in the same subject during the same extinction session. We found no difference in the proportion of extinguishing and non-extinguishing cells among sign trackers and goal trackers: sign trackers contributed a total of eight extinguishing and nine non-extinguishing cells, whereas goal trackers contributed 16 extinguishing cells and 19 non-extinguishing cells (*p* = 0.93, χ^2^ test). One cell showed a significant increase in cue-evoked firing and was not included in subsequent analyses.

Although the proportions of distinct neuronal response profiles were not different in sign trackers versus goal trackers, population cue-evoked activity across the extinction session was greater among sign trackers than goal trackers in extinguishing cells (*p* = 0.02, Wilcoxon rank-sum test; Fig. 6*C*) but not in non-extinguishing cells (*p* = 0.23; Fig. 6*D*) during the peak of excitation (0–300 ms after cue onset). If the tail of the excitation was included (0–500 ms or 0–1 s after cue onset), sign trackers exhibited greater average activity over the course of extinction among both cell types (all cases, *p* < 0.001). We hypothesized that this activity profile might result from a more gradual extinguishing of cue-evoked excitations among sign trackers than among goal trackers. Supporting this notion, when we examined average cue-evoked firing (0–500 ms after cue onset) in five-trial bins over the course of the extinction session (Fig. 6*E*), we observed that activity among extinguishing cells (solid lines) in sign trackers and goal trackers is initially indistinguishable (bin 1: *p* = 1, Wilcoxon rank-sum test) but then trends higher in sign trackers during trials 6–10 (bin 2: *p* = 0.09) before converging again. The same was not true for non-extinguishing cells (Fig. 6*E*, dashed lines). Thus, sign trackers exhibit a delayed extinction of cue-evoked activity relative to goal trackers that is mainly driven by a slower decline in activity among the subpopulation of extinguishing cells.

This slower decline in cue-evoked activity among sign trackers was paralleled by a more gradual decrease in ST behavior compared to GT behavior, as has been reported previously (Ahrens et al., 2016). Compared with magazine entries in goal trackers, the number of lever presses in sign trackers remains elevated later into the extinction session, as shown in Figure 6*F* (bin 2: *p* = 0.1, bin 4: *p* = 0.1, Wilcoxon rank-sum test); similarly, after starting out indistinguishable, the latency to first lever press after cue onset among sign trackers trends lower than latency to first magazine entry among goal trackers during trials 6–10 of extinction (bin 2: *p* = 0.1; Fig. 6*G*). Although the relatively small number of subjects precludes strong statistical conclusions about behavior, it is clear that the largest differences we observed in ST versus GT behavior occur at the same time as the largest differences in the decline of cue-evoked neural activity, consistent with the finding that cue-evoked firing encodes the vigor of both ST and GT.

In order to draw a more direct connection between the activity of individual cells and the extinction of behavior, we next examined the trial-by-trial correlation (Spearman’s rho) between firing rate in the 500 ms following cue onset and ST versus GT behaviors. Many individual correlations were significant (Extended Data Fig. 6-1), especially for goal-tracking behavior, which exhibited a larger dynamic range among subjects. Figure 6*H* shows the average correlation coefficient for the intensity (i.e., number) and latency of each behavior among sign trackers (blue) and goal trackers (magenta). Overall, neurons recorded in sign trackers had significantly higher correlation coefficients with ST behavior (number of lever presses: *p* = 0.03, Wilcoxon rank-sum test; latency to first lever press: *p* < 0.001), compared with neurons recorded in goal trackers. This finding held true when one goal tracker subject with zero lever presses was excluded. Conversely, neurons recorded in goal trackers had significantly higher correlation coefficients with goal-tracking behavior (number of magazine entries: *p* = 0.002; latency to first magazine entry: *p* = 0.02) than neurons recorded in sign trackers, even though all subjects, including sign trackers, displayed some degree of goal-tracking behavior during the extinction session.

10.1523/ENEURO.0414-18.2019.f6-1 Extended Data Figure 6-1 Correlation of the activity of individual neurons with behavioral extinction of ST and GT. Distribution of Spearman’s rank correlation coefficient (rho) relating cue-evoked neural activity (500-ms window) to number of lever presses (***A***, ***B***), number of magazine entries (***C***, ***D***), latency to first lever press (***E***, ***F***), or latency to first magazine entry (***G***, ***H***) for individual neurons recorded in sign trackers (***A***, ***C***, ***E***, ***G***) or goal trackers (***B***, ***D***, ***F***, ***H***) during an extinction session. All panels, blue indicates significant correlation (α = 0.1), and *p* values indicate results of Wilcoxon signed-rank test for median different from zero. Download Figure 6-1, EPS file.

Thus, among the subset of cells that extinguished their cue-evoked excitations during extinction, this activity decreased in concert with the subject’s predominant behavior, whether ST or GT, during the course of the session. This is consistent with the finding that many cue-evoked excitations reflect the vigor of the immediate subsequent action, whether lever press or magazine entry, on the final day of training (Fig. 3). Overall, these data support the hypothesis that the separable learning processes that produce ST and GT converge in the NAc to promote both forms of approach.

## Discussion

Individual animals show a wide range of behavior on a task in which a lever cue predicts the delivery of a reward in a separate location. Some animals are prone to transfer incentive salience to the cue, resulting in ST behavior (Hearst and Jenkins, 1974) – approach and/or interaction with the lever – whereas others animals are goal trackers: they tend to approach and/or interact with the site of reward rather than the cue (Boakes, 1977). The NAc plays an essential role in conditioned approach behaviors, including ST (Cardinal et al., 2002; Chang et al., 2012; although see Chang and Holland, 2013). In particular, dopamine release in the NAc is required for the acquisition and expression of ST, but not GT, behavior (Parkinson et al., 2002; Flagel et al., 2011; Saunders and Robinson, 2012; Fraser and Janak, 2017).

In the present study, we report both similarities and key differences between sign trackers and goal trackers in their patterns of NAc activity during the acquisition, maintenance, and extinction of ST and GT behavior. Cue-evoked excitations in the NAc encoded the vigor of the subsequent behavioral response, whether it was ST or GT, among subsets of recorded neurons. Meanwhile, although cue-evoked activity remained relatively stable over the course of training in all subjects, reward-evoked activity showed a marked decrease in sign trackers, but not goal trackers. Finally, during an extinction session, a subset of cue-excited neurons (extinguishing cells) decreased their activity in concert with behavior, a decrease that was more closely linked to lever presses among sign trackers, and to magazine entries among goal trackers. However, we observed an additional subset of NAc neurons (non-extinguishing cells) that did not decrease their cue-evoked activity over the course of behavioral extinction.

### Convergence of multiple forms of reward learning in the NAc

Consistent with prior studies using both Pavlovian tasks (Day et al., 2006) and instrumental tasks (McGinty et al., 2013), we found that a large proportion of NAc neurons (averaging ∼58%) exhibit excitatory responses to cues that are associated with reward. These cue-evoked excitations have been shown to encode the vigor of subsequent locomotor responses, e.g., approach to a reward-associated lever, including such factors as latency and speed, as well as the probability that a behavioral response will occur at all (McGinty et al., 2013; Morrison and Nicola, 2014; Morrison et al., 2017). Interestingly, this encoding is much more prominent during tasks that require taxic approach – i.e., in which the cue elicits a novel action sequence – rather than praxic approach, in which the cue elicits one of a limited subset of possible actions (McGinty et al., 2013). Indeed, NAc activity, as well as dopaminergic function, is specifically required for taxic but not praxic approach tasks (Nicola, 2010).

Both ST and GT behavior require taxic approach towards a reward-associated target – either the lever or the food magazine – so, in that regard, we might expect that the vigor of both behaviors would be represented in NAc cue-evoked activity. Indeed, we found that many individual neurons have stronger cue-evoked firing when the subsequent behavioral response, whether lever press or magazine entry, occurred with shorter latency. In fact, despite the special importance of the NAc for the acquisition and expression of PCA, including ST (Cardinal et al., 2002), the relationship of cue-evoked firing to the vigor of GT (represented by latency to enter the food magazine) was particularly strong relative to ST. This might be a consequence of the larger dynamic range of goal-tracking behavior both within subjects and between subjects: GT was present in all subjects to some degree, whereas ST behavior was exhibited by only the subset of subjects categorized as sign trackers.

It is important to note that the essential role of the NAc, especially NAc dopamine release, in ST is not incompatible with a role for accumbens neuronal activity in GT. Although few studies have directly compared the impact of loss of NAc function on ST versus GT, it has been shown that lesion (Parkinson et al., 1999) or reversible inactivation (Blaiss and Janak, 2009) of the NAc core impairs the expression of goal-tracking behavior, at least to a moderate extent, during Pavlovian conditioning tasks in which GT is the primary response. Notably, however, inactivation of the NAc does not impair the initial acquisition of goal-tracking behavior (Blaiss and Janak, 2009). In contrast, a number of studies have shown that a functional NAc is necessary for the acquisition of ST and other forms of PCA (Di Ciano et al., 2001; Dalley et al., 2005; Chang et al., 2012; but see Chang and Holland, 2013). The idea that the NAc is specifically involved in the acquisition of ST, but plays a role in the expression of both ST and GT, is in line with our finding that the learning processes underlying ST versus GT are reflected by differently evolving activity patterns in the NAc.

Finally, the current evidence that NAc cue-evoked activity promotes the vigor of both ST and GT supports the notion that the NAc functions as a node of interaction between different forms of reward learning (Clark et al., 2012; Lesaint et al., 2014). Mounting evidence indicates that ST arises from a dopamine-dependent form of learning that results in the transfer of incentive value from reward to cue and is relatively independent of the sensory characteristics of the outcome, at least under some conditions (Flagel et al., 2011; Clark et al., 2012; Huys et al., 2014; Morrison et al., 2015; although see Derman et al., 2018). GT, on the other hand, is thought to arise from a dopamine-independent form of learning that incorporates sensory characteristics of the outcome, as it is profoundly sensitive to manipulations of outcome value (Morrison et al., 2015) or cue-outcome relationship (Beckmann and Chow, 2015; Ahrens et al., 2016). These disparate learning processes appear to converge in the accumbens, supporting the idea that a key function of the NAc is to invigorate approach towards reward-associated targets (Morrison and Nicola, 2014), regardless of the source of the stimulus-reward association.

### Relationship of NAc single-unit activity to phasic dopamine release

It has been shown that sign trackers and goal trackers, whether selectively bred “high responders” and “low responders” or outbred rats, exhibit different characteristic patterns of NAc dopamine release during training on a PCA task comparable to the one used here. Using fast-scan cyclic voltammetry, Flagel et al. (2011) found that, on average, sign trackers showed increased dopamine release in response to the cue, and decreased dopamine release in response to the reward, over the course of six training sessions. Goal trackers, on the other hand, showed relatively stable levels of dopamine release in response to the cue and reward throughout training. These results implied that sign trackers, but not goal trackers, were utilizing the reward prediction error encoded by phasic dopamine (Waelti et al., 2001) as a teaching signal, consistent with the notion that ST, but not GT, is a manifestation of dopamine-dependent reinforcement learning.

In the current study, we demonstrate that the differences between sign trackers and goal trackers in patterns of NAc dopamine release are at least partially reflected by the task-related activity of single neurons in the NAc. Over the course of training, even during the very first training session, sign tracker individuals exhibit a marked decrease in neuronal firing evoked by reward delivery, whereas goal tracker individuals do not. This finding mirrors the decrease in reward-evoked NAc dopamine release seen in sign trackers, but not goal trackers, during learning (Flagel et al., 2011), and supports the idea that, among sign trackers only, the motivational value of the reward undergoes a transfer from the reward itself to the predictive cue.

On the other hand, in contrast to the increase in cue-evoked phasic dopamine seen in sign trackers (Flagel et al., 2011), we observed little to no change in neural activity in response to the reward-predictive lever cue. Among goal trackers only, there was a small increase in cue-evoked activity over the course of the first training session; but there was no significant difference in population activity between the first and last sessions for either sign trackers or goal trackers. There are at least two possible reasons for this discrepancy. The first is that, in the current study, operationally defined sign trackers all performed an appreciable amount of GT behavior in addition to ST. Indeed, sign trackers executed more magazine entries than goal trackers during the first 2 d of training (Fig. 1*D*), and their level of GT stayed relatively stable throughout training, even as their ST behavior increased. Because cue-evoked excitations represent the vigor of GT more robustly than that of ST in the current dataset, it is perhaps not surprising that sign trackers’ cue-evoked firing remained stable throughout the acquisition period.

Second, Flagel et al. (2011) find that, among outbred sign trackers, the increase in cue-evoked phasic dopamine release is relatively modest compared with the robust decrease in reward-evoked dopamine release. This is consistent with our finding of a strong decrease in reward-evoked firing among sign trackers along with a small, non-significant increase in cue-evoked firing. It has been shown that activation of D1 and/or D2 dopamine receptors in the NAc enhances cue-evoked excitatory responses (du Hoffmann and Nicola, 2014), so we might expect that sign trackers’ increase in cue-evoked dopamine release over the course of training would result in increased cue-evoked neuronal activity. However, any additional firing resulting from a small increase in phasic dopamine release – i.e., as part of a dopamine-dependent learning process – may be rendered undetectable by the already-strong cue-evoked excitation, perhaps resulting from a concurrent non-dopamine-dependent process that promotes vigorous GT responses.

Indeed, it is important to note that goal trackers, as well as sign trackers, exhibit dopamine release in response to reward predictive cues, even though acquisition of GT behavior does not depend on NAc dopamine (Flagel et al., 2011; Saunders and Robinson, 2012). This observation is consistent with the idea that phasic mesolimbic dopamine release plays a dual role: invigorating action directed towards reward-associated targets in addition to facilitating simple forms of reinforcement learning (Guitart-Masip et al., 2012; Ko and Wanat, 2016; Syed et al., 2016; Berke, 2018). Although the precise relationship between sub-second dopamine release and neuronal firing in target regions has been difficult to determine, we would speculate that the cue-evoked excitations we observe in both sign trackers and goal trackers more strongly reflect the former function of dopamine, action invigoration, whereas the decreasing reward-evoked responses observed in sign trackers reflect the latter function, reinforcement learning.

Finally, we found that, among both sign trackers and goal trackers, the large majority of cue-excited NAc neurons also exhibit excitatory responses to reward delivery. This result stands in apparent contrast with the frequently reported finding that consummatory actions are accompanied by inhibition of neuronal activity in the NAc (Nicola et al., 2004; Taha and Fields, 2005; Wan and Peoples, 2006; Roitman et al., 2010). Although a small subset of NAc neurons encode the value of a reward via excitatory responses during consumption (Taha and Fields, 2005), we believe it is more likely that the brief excitations we observe are occurring prior to actual consumption. Rather, they may be related to the sight and/or sound of the sucrose pellet dropping into the food magazine – i.e., by cues conveying the information that reward has been delivered – rather than to the hedonic experience of sucrose consumption or to consummatory actions such as chewing. Indeed, although we did not track consummatory behavior in the current study, excitations associated with reward delivery were often followed by inhibitions, which were likely associated with pellet consumption. Notably, this profile of reward-related NAc activity roughly corresponds, in both direction and scale, to the time course of NAc dopamine release in response to delivery of a sucrose pellet following a reward-predictive cue (McCutcheon and Roitman, 2018).

### Divergent profiles of NAc activity during behavioral extinction

Previous studies have shown that ST behavior is relatively resistant to extinction, compared with goal-tracking behavior, both within subjects (Beckmann and Chow, 2015) and between subjects (Ahrens et al., 2016). This is likely the result of sign trackers’ tendency to attribute incentive salience to the cue, resulting in continued cue-directed actions even in the absence of reward. In support of this idea, a lever cue is much more effective as a conditioned reinforcer in sign trackers than in goal trackers (Robinson and Flagel, 2009), indicating that the cue has been imbued with motivational value. On the other hand, sign trackers and goal trackers do not differ in their rates of instrumental extinction (Yager and Robinson, 2010; Ahrens et al., 2016), implying that sign trackers’ dopamine-dependent learning system is selectively and preferentially engaged during Pavlovian conditioning.

In the current study, we confirm that ST behavior (among sign trackers) extinguishes more slowly than GT (among goal trackers). Further, we demonstrate that the cue-evoked excitatory responses of many neurons in the NAc decrease, or extinguish, in concert with behavior: these extinguishing cells decrease their firing more rapidly in goal trackers than in sign trackers, on average. Finally, we show that the decreasing cue-evoked response is more closely associated with decrements in lever pressing among sign trackers, and with decrements in magazine entry among goal trackers. All of these findings are consistent with the notion that NAc cue-evoked excitations invigorate approach towards reward-associated targets, regardless of the source of the association or the specific form of the conditioned response, and that a reduction in NAc firing elicited by a cue will increase the latency and decrease the probability of a behavioral response to that cue (Morrison et al., 2017).

Although no study, to our knowledge, has compared dopamine release in sign trackers and goal trackers during extinction, our observation that NAc activity gradually extinguishes when reward is no longer available is consistent with the finding that cue-evoked phasic dopamine release decreases over the course of Pavlovian extinction (Sunsay and Rebec, 2014). At least among extinguishing cells in the NAc, it is likely that dopamine release acts as a gating mechanism permitting cue-evoked firing and, as a result, behavioral responding to the cue (du Hoffmann and Nicola, 2014). This gradual decrease in both dopamine release and cue-evoked NAc firing could provide a neural substrate for the kind of “unlearning” process of extinction posited by traditional reward prediction error models of reinforcement learning (Rescorla and Wagner, 1972; Schultz et al., 1997).

On the other hand, it is now widely recognized that extinction involves more than unlearning: phenomena such as reinstatement and spontaneous recovery demonstrate that the original cue-reward association is not forgotten and may be retrieved in a different context or after the passage of time (Todd et al., 2014). Consistent with this idea, in addition to extinguishing cells, we observed almost equal numbers of non-extinguishing cells: NAc neurons with cue-evoked excitatory responses that do not decrease over the course of behavioral extinction. The proportions of these cells did not differ between sign trackers and goal trackers, whose different rates of behavioral extinction might be better explained by divergent reductions in cue-evoked firing among extinguishing cells only. Rather, non-extinguishing cells might constitute part of the neural circuitry that maintains a latent representation of the cue-reward relationship following extinction. Interestingly, their cue-evoked responses appear to be resistant to the decrease in phasic dopamine release that accompanies extinction (Sunsay and Rebec, 2014). Further investigations will be necessary to determine whether these non-extinguishing cells differ from extinguishing cells in characteristics such as dopamine receptor expression and/or participate in anatomically separable circuits. If so, extinguishing and non-extinguishing cells could provide a novel neural substrate for the simultaneous new learning and maintenance of prior associations that characterizes extinction (Pan et al., 2008; Todd et al., 2014).

Overall, we observed both similarities, such as robust encoding of food magazine-directed behavior, as well as key differences between sign trackers and goal trackers in NAc neuronal activity patterns, including a decrease in reward-related activity specific to sign trackers that appears to reflect reward prediction error signals encoded by phasic dopamine. Indeed, these findings highlight the widely varying extent to which phasic dopamine, as a signal, is reflected in the neuronal activity of target structures. This is certainly true of NAc cue-evoked activity during extinction, which broadly reflects decreases in phasic dopamine release, but also includes non-extinguishing cells that do not decrease their activity in concert with dopamine release and behavior. Ultimately, understanding how differences in dopamine release are translated into neural activity will provide insight into how and why different individuals – e.g., sign trackers and goal trackers – engage different learning systems (Clark et al., 2012; Huys et al., 2014; Lesaint et al., 2014) when cues in the environment predict reward.

## Synthesis

Reviewing Editor: Mark Baxter, Mount Sinai School of Medicine Decisions are customarily a result of the Reviewing Editor and the peer reviewers coming together and discussing their recommendations until a consensus is reached. When revisions are invited, a fact-based synthesis statement explaining their decision and outlining what is needed to prepare a revision will be listed below. The following reviewer(s) agreed to reveal their identity: Kurt Fraser.

We were impressed with the quality of the manuscript and find the approach and results very interesting. Despite previous papers similarly exploring these issues (Wan & Peoples 2006, Day, Wheeler, Roitman & Carelli 2006), the authors attempt to make use of a task design that allows for the identification of divergent conditioned responses, goal- and sign-tracking. This is of great interest as dopamine in the nucleus accumbens core is essential for the development and expression of sign-tracking but not goal-tracking. In contrast, inactivation or lesion of the nucleus accumbens core in sign- and goal-tracking rats does not have any evident effect on behavior (Fraser & Janak 2017, Chang, Wheeler, & Holland 2012, Blaiss & Janak 2009), suggesting that maybe neural activity isn't essential in this region but dopaminergic modulation is. To explore this, the authors trained rats in a modified version of the autoshaping task commonly used by Robinson and colleagues, where a lever, accompanied by a light and auditory cue, was present for 8s, and immediately followed by food pellet delivery. Critically, rats were implanted with chronic electrodes prior to training to allow for the recording of neural activity across the 7 days of training in the task, which is how long it has been documented for behavior to become stable.

Overall, the main concerns about the manuscript relate to the classification of rats into ‘sign-trackers’ and ‘goal-trackers’ based on traditional criteria - only one rat preferentially shows sign-tracking behavior (PCA index &gt; 0) - and whether the analyses could be elaborated. Although there is some concern about whether additional analyses would provide unambiguous results, we think a revised manuscript that addresses these concerns could be prepared with less than 2 months additional work.

Major concerns:

1) The authors do not appear to have any traditionally and acceptably defined sign-trackers in the sample of rats who were recorded. This makes all claims in the paper difficult to interpret and hard to link with existing literature. Additionally, there aren't enough recorded neurons that are cue-responsive in each group of identified subjects that are sufficient for these analyses. When cue responses are sampled in sign- and goal- trackers it is often unclear what the response is. On day 1 for example, most responses are goal-tracking, while later there is more of a mix (at least for sign trackers). It appears as though sign-trackers do both to some extent, and also generally have a greater number of overall responses (adding goal entries and lever presses together). Thus it would be important to understand how the cue (and even reward) firing responses relate to what the actual behavioral response is. Does it relate to sign-tracking itself, or goal-tracking itself, or a mix? This might require shorter time windows of analysis to address, but would be a highly useful addition because at the moment the per-trial behavioral response itself seems to be overlooked in favor of the group division but could potentially contribute to the results that are presented. On this point, correlation/regression analyses on per-trial behavioral responses and firing rates would be highly useful to include for all results sections because the extent of sign-tracking appears to be a sliding scale. The sign/goal division in groups is informed and helpful for analysis but seems to be an illusory division to make judging by the figures. Building analysis around the actual behavior as it occurs rather than a group division may make much more sense in this case.

2) Despite the use of chronic electrodes, the authors do not make use of waveform similarity analyses to provide evidence that they are holding the same sets of neurons across days. The addition of such analyses would greatly strengthen the manuscript and allow for stronger claims about the development of task-relevant responses in the nucleus accumbens core.

3) The extinction test is a great approach, but the test is conducted on a separate day after the end of training. This required unplugging and replugging rats, and as noted above the authors are not able to identify stably recorded units across sessions. Is there a justification for the extinction test not immediately follow the final training session to overcome these limitations?

4) As opposed to the vigor index, the authors should correlate the magnitude of neural response to cue-onset and the latency to begin magazine or lever-directed responding. Do the authors also have video-taped recordings of behavior to additionally correlate responses to the onset of movement to either the lever or magazine?

5) A rationale should be provided for why analyses were done on relatively long 1 sec or 0.5 sec bins, and why one or the other bins were selected for different analyses. There seems to be differences in firing responses at different points within these bins.

6) The authors could include a more thorough analysis of baseline activity differences between groups and conditions (e.g. via z-score normalization of full session activity perhaps). Changes in baseline activity would affect any normalized event-related activity measure.

7) We could not find statistical comparisons that support that there are “clear differences between sign- and goal-trackers in the evolution of NAc activity” as in Figure 4 there is no change in cue-responses in this session for either group of rats, nor could I find direct comparisons between the neural activity in sign- and goal-trackers. Did the authors include phenotype as a factor in these analyses? (This may be less of a concern if analyses focus on correlations with trial-by-trial behavior instead of on a split between sign-tracker and goal-tracker rats.)

Minor concerns:

1) The authors should include trial-by-trial behavioral data for the data described in Figure 6.

2) The relationship between cells that are modulated by latency to how they are modulated by extinction learning is excellent, but the same relationship is not as well explored for other sections of the results. It would be nice to flesh that out.

3) It would be helpful to address the question of whether cue-responsive cells are also reward-responsive, or not, and if a decline in reward-responsiveness is in the same neurons that respond to cues, or not.

4) In the discussion, the authors should consider that the effects of NAc core inactivations on goal-tracking are not very profound (Blaiss & Janak 2009) especially in comparison to the effects of dopamine antagonism on sign-tracking and the lack of inactivation effects on sign-tracking behavior (Fraser & Janak 2017). Chang & Holland (Behav Brain Res 2013) show that the NAc core is not required for sign tracking. This is opposed to general role for NAc in vigor and the expression of these responses as the authors suggest.

5) The authors note that sign tracking is related to impulsivity. Are the findings of Belin et al Science 2008 relevant?

6) The model-based/model-free distinction is presented without much context and is a bit confusing. Isn't sign-tracking immediately sensitive to appetite states and not just model-free then? (Robinson Berridge Curr Biol 2013).

7) The authors are free to use ‘PCA’ but it is confusing given the more common use of this acronym is for principal components analysis.
